# Understanding the molecular basis of folding cooperativity through a comparative analysis of a multidomain protein and its isolated domains

**DOI:** 10.1016/j.jbc.2023.102983

**Published:** 2023-02-03

**Authors:** Daniele Santorelli, Lucia Marcocci, Valeria Pennacchietti, Caterina Nardella, Awa Diop, Paola Pietrangeli, Livia Pagano, Angelo Toto, Francesca Malagrinò, Stefano Gianni

**Affiliations:** 1 Dipartimento di Scienze Biochimiche “A. Rossi Fanelli”, Istituto Pasteur-Fondazione Cenci Bolognetti and Istituto di Biologia e Patologia Molecolari del CNR, Sapienza Università di Roma, Rome, Italy; 2 Dipartimento di Farmacia, Università degli Studi di Napoli Federico II, Naples, Italy

**Keywords:** kinetics, multidomain folding, cooperativity, protein stability, PDZ domains, ANS, 8-anilino-1-naphthalenesulfonic acid, CD, circular dichroism

## Abstract

Although cooperativity is a well-established and general property of folding, our current understanding of this feature in multidomain folding is still relatively limited. In fact, there are contrasting results indicating that the constituent domains of a multidomain protein may either fold independently on each other or exhibit interdependent supradomain phenomena. To address this issue, here we present the comparative analysis of the folding of a tandem repeat protein, comprising two contiguous PDZ domains, in comparison to that of its isolated constituent domains. By analyzing in detail the equilibrium and kinetics of folding at different experimental conditions, we demonstrate that despite each of the PDZ domains in isolation being capable of independent folding, at variance with previously characterized PDZ tandem repeats, the full-length construct folds and unfolds as a single cooperative unit. By exploiting quantitatively, the comparison of the folding of the tandem repeat to those observed for its constituent domains, as well as by characterizing a truncated variant lacking a short autoinhibitory segment, we successfully rationalize the molecular basis of the observed cooperativity and attempt to infer some general conclusions for multidomain systems.

Despite multidomain proteins characterize about 75% of the human proteome, our current understanding of the general rules of protein folding is primarily based on studies on single domain proteins (1). In fact, complex proteins are more difficult to address experimentally and are therefore generally very elusive to a detailed depiction. Moreover, the capability to express and characterize protein domains in isolation has led to the general assumption that they are able to fold and function independently (2, 3, 4, 5, 6, 7, 8). However, the transient interaction between these structural subunits may be responsible for complex effects that demand a careful investigation. For example, a number of works have demonstrated the simultaneous denaturation of two contiguous domains to be responsible for the transient accumulation of misfolded intermediates (6, 9, 10, 11, 12, 13). These effects are more pronounced when the sequence homology of contiguous domains is relatively high, typically more than 40%, a condition that may promote transient domain swapping (14).

While transient misfolding seems to represent a recurring and well-established phenomenon in multidomain proteins, present knowledge on observed cooperativity appears much more elusive. While very frequently isolated protein domains fold well and cooperatively in isolation, there is sparse indication that some multidomain constructs fold and unfold as single cooperative unit (14, 15, 16, 17), thereby questioning the nature of the thermodynamic interactions between these structural subunits. At present there are no general rules to predict the independent folding of individual domains as opposed to super-tertiary cooperative (un)folding.

A recurrent type of multidomain organization in proteins is represented by tandem repeats (18, 19, 20). In these cases, structurally homologous domains are presented in a contiguous assembly. We have recently characterized the folding of two different tandems comprising a repetition on two PDZ domains and, in both cases, found the folding of each domain to occur independently of the other, aside from transient misfolding events (8, 11). In an effort to extend our comparative work on tandem repeats, we present here the extensive kinetic and equilibrium characterization of the folding of the tandem PDZ1-PDZ2 from X11, also comprising two homologous PDZ domains (21). As detailed below, at variance with the previous examples, we found that X11 PDZ1-PDZ2 displays a remarkable cooperativity and folds and unfold as a single unit. By taking advantage of the comparison of the folding of the tandem repeat to those observed for its constituent domains, as well as by characterizing a truncated variant, lacking nine amino acids that constitute an autoinhibitory segment (21), we successfully rationalize the molecular basis of the observed cooperativity of X11 PDZ1-PDZ2 and attempt to draw some general conclusions on multidomain folding.

## Results

An insightful approach to address the folding of tandem repeats is to study their behavior in comparison to what observed for its constituent isolated domains (1, 9, 11, 17). Accordingly, the experimental results reported below will be presented to emphasize the major differences and similarities between the different constructs. Finally, we will describe some results obtained on a truncated variant of X11 PDZ1-PDZ2, which are critical to allow some conclusions on the molecular basis of the folding of this tandem repeat.

### The folding and unfolding of X11 PDZ1-PDZ2 in comparison to its isolated constituent domains

**Figure 1:**
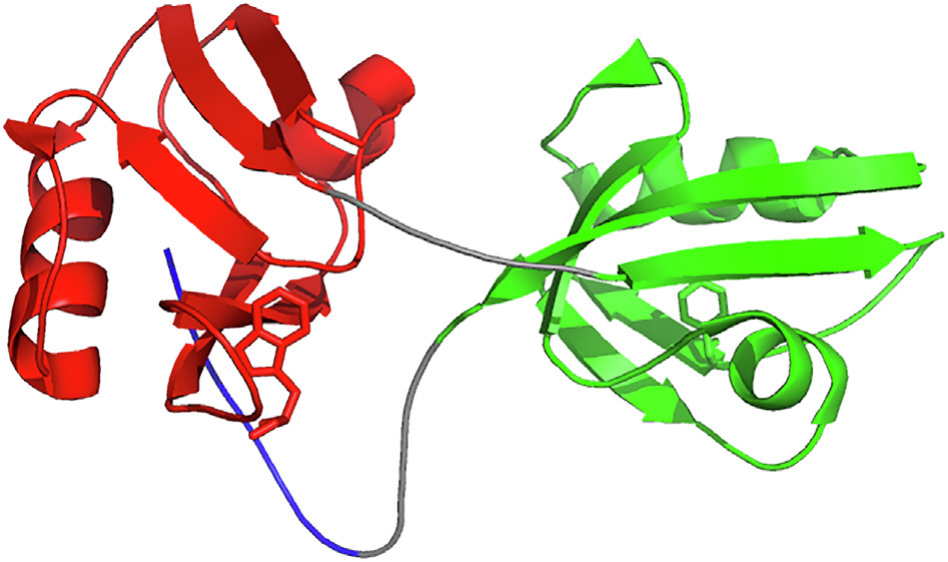
**The structure of X11 PDZ1-PDZ2 (pdb****1U3B****).** PDZ1 and PDZ2 are highlighted in *red* and *green* respectively. The autoinhibitory C-terminal segment comprising the last nine amino acids is depicted in *blue*. Trp676 in PDZ1 and Phe761 in PDZ2 are highlighted in *sticks*.

X11 PDZ1-PDZ2 is a tandem repeat comprising two PDZ domains (Fig. 1), namely PDZ1 and PDZ2 (21). While PDZ1 contains a Trp residue in position 676, in the case of PDZ2 no fluorescent probe is available.

**Figure 2:**
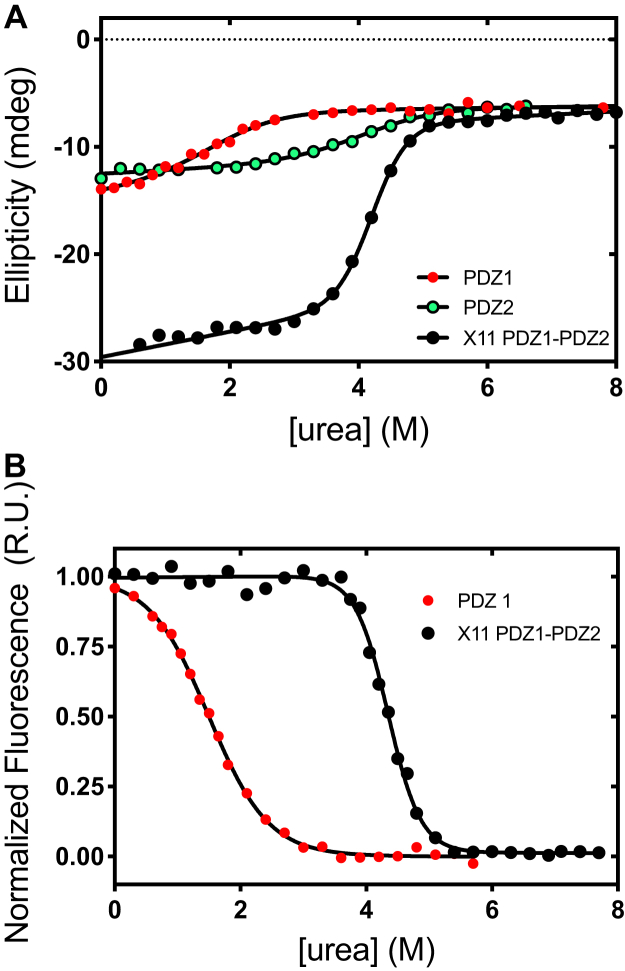
**Equilibrium denaturation of X11 PDZ1-PDZ2 and its constituent isolated domains.***A,* CD-monitored equilibrium denaturation of X11 PDZ1-PDZ2, *black circles*, PDZ1*, red circles*, and PDZ2, *green circles*. *Lines* are the best fit to a two-state transition. *B*, fluorescence-monitored equilibrium unfolding of X11 PDZ1-PDZ2, *black circles*, and PDZ1, *red circles*. As discussed in the text, there was an excellent agreement between the thermodynamic parameters calculated for both PDZ1 and X11 PDZ1-PDZ2 when comparing CD and fluorescence. As detailed in the text, in the case of PDZ2, we could not detect any reliable transition due to the absence of Trp residues. *Lines* are the best fit to a two-state unfolding transitions. It is evident that, both by fluorescence and CD, the observed cooperativity of X-11 PDZ1-PDZ2 is remarkably higher than that observed for its isolated constituent domains. CD, circular dichroism.

The circular dichroism (CD) monitored equilibrium (un)folding of X11 PDZ1-PDZ2 in comparison to that monitored for PDZ1 and PDZ2 in isolation is reported in Figure 2. In all cases, we observed a sigmoidal transition consistent with a two-state behavior (22). Notably, however, in the case of X11 PDZ1-PDZ2, observed cooperativity appears much more pronounced than that observed for its constituent isolated domains, suggesting that this molecule behaves a single cooperative unit. A quantitative analysis of the observed transition returned an *m*_D–N_ value of 2.1 ± 0.2 kcal mol^−1^ M^−1^ with a *ΔG*_*D–N*_ of 8.8 ± 0.9 kcal mol^−1^. By considering that the *m*_D–N_ value, which is the slope of the dependence of the free energy of unfolding *versus* denaturant concentration, represents a measure of the change in accessible surface area upon unfolding (23), it is interesting to observe that, while PDZ1 and PDZ2 return a value which is consistent with a domain of about 90 amino acids, the full length X11 PDZ1-PDZ2 displays a nearly doubled *m*_D–N_ value, with PDZ1 and PDZ2 returning an *m*_D–N_ value of 1.0 ± 0.1 kcal mol^−1^ M^−1^ and 0.95 ± 0.08 kcal mol^−1^ M^−1^, respectively. This observation clearly indicates that the latter construct behaves a single cooperative unit. In fact, if the two domains were displaying a concurrent noncoperative denaturation, while a single transition could be observed, the apparent *m*_D–N_ value would appear much lower than the observed value of 2.1 ± 0.2 kcal mol^−1^ M^−1^. This hypothesis would also appear unlikely given the different stability of the two distinct domains. To confirm that the two PDZ domains maintain a similar secondary structure when considered in isolation, we compared the CD spectrum X11 PDZ1-PDZ2 to that obtained by mixing the individual PDZ1 and PDZ2 at equimolar concentration. The resulting spectra, reported in Fig. S1, were nearly superposable, confirming that the two PDZ domains fold well in isolation.

The fluorescence-monitored transitions are reported in Figure 2*B*. Since no fluorescent residue is present in PDZ2, no transition could be observed in this case. A quantitative analysis of the data obtained for PDZ1 and X11 PDZ1-PDZ2 returned 2.1 ± 0.1 kcal mol^−1^ M^−1^ with a *ΔG*_*D–N*_ of 9.1 ± 0.4 kcal mol^−1^. These values are consistent with those measured by CD, confirming the two-state nature of the equilibrium transition in both cases, as well as the remarkably higher cooperativity observed in the case of the X11 PDZ1-PDZ2 tandem.

### The folding and unfolding rate constants of X11 PDZ1-PDZ2 at high denaturant and in water are limited by PDZ1

To provide a complete description of the folding and unfolding reactions of X11 PDZ1-PDZ2 in comparison to its constituent domains, we performed kinetic experiments at a variety of different experimental conditions. As outlined above, while a Trp residue is present in PDZ1 at position 676, no fluorescence probe is present in PDZ2. Hence, for the sake of clarity, we will first describe the observed kinetics of folding of X11 PDZ1-PDZ2 with PDZ1 and subsequently analyze the behavior of PDZ2.

**Figure 3:**
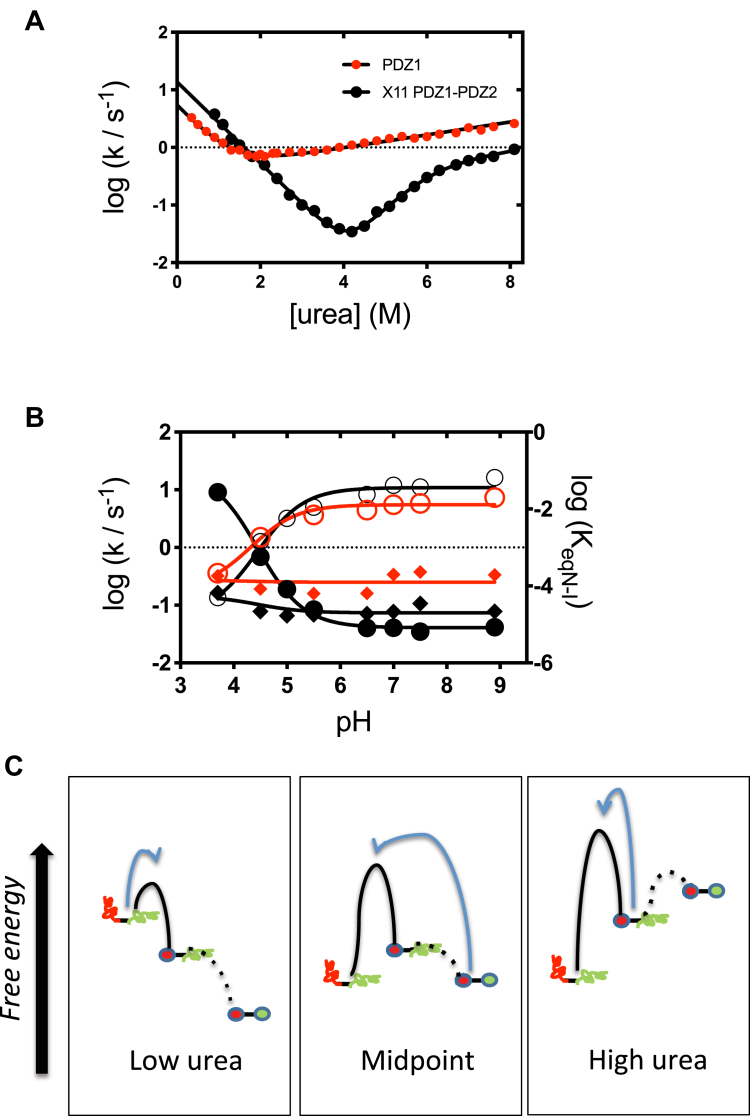
**Folding kinetics of X11 PDZ1 and PDZ2.***A,* the chevron plots of X11 PDZ1-PDZ2, *black circles*, and PDZ1, *red circles*. It is evident that, while X11 PDZ1-PDZ2 presents a pronounced curvature in the unfolding branch, PDZ1 conforms to a V-shape chevron plot. Accordingly, the two different constructs were fitted to a three-state and a two-state model, respectively. *Lines* are the best fit obtained for the two proteins. *B,* dependence of the folding and unfolding rate constants of X11 PDZ1-PDZ2 and PDZ1 on pH. The folding rate constants *k*_F_ are represented in *open circles*; unfolding rate constants *k*_U_ are represented in *diamonds*, whereas the stability of the folding intermediate as probed by the *K*_eqN–I_, which could be identified for X11 PDZ1-PDZ2 only, is represented in *filled circles*. By following a three-state analysis of the observed chevron plots, the *K*_eqN–I_, value was estimated in the absence of denaturant, and the calculated values are reported in the right-y axis. Parameters referring to X11 PDZ1.PDZ2 are reported in *black*, whereas those referring to PDZ1 are reported in *red*. As discussed in the test, it is evident that X11 PDZ1-PDZ2 and PDZ1 display a very similar pH dependence of the folding and unfolding rate constants, indicating that at very high and low denaturant concentrations, (un)folding is limited by PDZ1. This finding is further supported by the conserved m values for these two parameters at different experimental conditions (see text and Fig. S1). *C*, a plausible energy diagram describing the reaction mechanism of (un)folding of X11 PDZ1-PDZ2. PDZ1 is depicted in *red*, whereas PDZ2 is depicted in *green*. The diagram schematically explains how both folding at low denaturant concentrations and unfolding at very high denaturant concentrations are limited by PDZ1. By following this view, the folding intermediate should involve the presence of folded PDZ1 with PDZ2 by-and-large denatured.

The folding and unfolding kinetics of both X11 PDZ1-PDZ2 and PDZ1 were investigated at several pH values ranging from 3.7 to 9 and at 37 °C. In all cases, the observed time courses for both folding and unfolding experiments could be satisfactorily fitted to a single exponential decay at any final denaturant concentration. A semi-logarithmic plot of the observed folding/unfolding rate constant *versus* denaturant concentration, commonly denoted as ‘chevron plot’, measured at neutral pH value is reported in Figure 3*A*. While in the case of PDZ1, the chevron plot conforms to a simple V-shape, a hallmark of two-state folding (22), and in the case of X11 PDZ1-PDZ2, it displays additional complexity. In fact, the latter construct presents a downward curvature at high urea concentrations (roll-over effect), which represents a signature for the presence of a folding intermediate (24, 25, 26).

To address quantitatively the folding and unfolding kinetics of X11 PDZ1-PDZ2 and PDZ1, the chevron plots measured at different pH values (Fig. S2) were fitted globally with shared kinetic m-values by following a three state and a two state model, as formalized in the Methods section, respectively, for the two different constructs. Kinetic folding parameters obtained from quantitative chevron analysis are reported in Table 1. Notably, while two-state folding was applicable for PDZ1, at all different experimental conditions, X11 PDZ1-PDZ2 displayed a pronounced roll-over in its unfolding branch, further confirming the presence of a transiently populated intermediate. Remarkably, in both cases, the thermodynamic parameters derived from equilibrium and kinetic experiments were in good agreement, reinforcing the reliability of our quantitative analysis.

**Table 1:** Kinetic folding parameters of X11 PDZ1-PDZ2 and PDZ1

pH	*k*_F_ (s^−1^)	*k*_U_ (s^−1^)	*K*_N–I_
X11 PDZ1-PDZ2			
3.7	0.14 ± 0.02	0.16 ± 0.02	(2.72 ± 0.08) •10^−2^
4.5	1.30 ± 0.08	0.078 ± 0.003	(5.7 ± 0.1) •10^−4^
5.5	5.06 ± 0.05	0.068 ± 0.002	(2.45 ± 0.04) •10^−5^
6.5	8.32 ± 0.05	0.071 ± 0.001	(8.12 ± 0.02) •10^−6^
7.0	12.0 ± 0.1	0.077 ± 0.002	(8.19 ± 0.06) •10^−6^
7.5	11.2 ± 0.1	0.107 ± 0.003	(6.53 ± 0.09) •10^−6^
9.0	16.3 ± 0.1	0.077 ± 0.001	(8.47 ± 0.04) •10^−6^
PDZ1			
3.7	0.36 ± 0.05	0.32 ± 0.02	-
4.5	1.51 ± 0.04	0.19 ± 0.05	-
5.5	3.7 ± 0.04	0.16 ± 0.06	-
6.5	4.5 ± 0.05	0.16 ± 0.07	-
7.0	5.5 ± 0.03	0.34 ± 0.06	-
7.5	5.8 ± 0.07	0.38 ± 0.08	-
9.0	7.3 ± 0.06	0.33 ± 0.05	-

Folding parameters obtained for X11 PDZ1-PDZ2 and PDZ1 were calculated from a three-state and two-state, respectively. All data were recorded at 37 °C. To reduce errors, the chevron plots of each protein were globally fitted with shared *m*-values. For X11 PDZ1-PDZ2, the calculated *m* values from a three-state analysis were *m*_F_ = 0.94 ± 0.03 kcal mol^−1^ M^−1^; *m*_U_ = 0.15 ± 0.02 kcal mol^−1^ M^−1^; *m*_KN–I_ = 1.09 ± 0.09 kcal mol^−1^ M^−1^, with a total *m*_D–N_ = 2.2 ± 0.1. This value is in good agreement with the value of 2.1 ± 0.2 kcal mol^−1^ M^−1^ obtained from CD monitored denaturation experiments. On the other hand, in the case of PDZ1, a two-state fit returned the values of *m*_F_ = 1.04 ± 0.04 kcal mol^−1^ M^−1^ and an *m*_F_ = 0.15 ± 0.01 kcal mol^−1^ M^−1^ with a total *m*_D–N_ = 1.2 ± 0.1 kcal mol^−1^ M^−1^. Also in this case, there is a good agreement with the equilibrium value monitored by CD melting, being *m*_D–N_ = 1.0 ± 0.1 kcal mol^−1^ M^−1^.

It is of interest to compare the dependence of the calculated folding and unfolding rate constants of X11 PDZ1-PDZ2 and PDZ1 on pH (Fig. 3*B*). In fact, although the observed difference in the kinetic mechanism, with a shift from two-state in the case of PDZ1 to a three state in the case of X11 PDZ1-PDZ2, both the folding rate constant *k*_F_ and the unfolding rate constant *k*_U_ display a very similar dependence. More to the point, the respective *m* values associated with the two rate constants were highly conserved at all investigated conditions. This finding may be qualitatively appreciated by comparing the slope the unfolding branch of PDZ1 with that observed at high denaturant concentrations for X11 PDZ1-PDZ2, as well as that of the refolding branches, which are very similar in entire data set reported in Fig. S1. On the basis of these observations, since at all the investigated conditions the folding of X11 PDZ1-PDZ2 is limited by PDZ1, it may be concluded that the folding and unfolding rate constants of PDZ1 are most likely the rate limiting step for the (un)folding of X11 PDZ1-PDZ2 tandem at very low and very high denaturant concentrations.

A plausible energy diagram describing the folding mechanism of X11 PDZ1-PDZ2 based on these considerations is reported in Figure 3*C*. At variance with two-state equilibrium, the analysis of the folding kinetics of X11 PDZ1-PDZ2 reveals the presence of a metastable intermediate, which accumulates transiently at high denaturant concentrations. Importantly, the folding rate constant of X11 PDZ1-PDZ2 as well as its unfolding rate constant at high denaturant concentrations, *i.e.*, when the intermediate is accumulated, display a nearly identical *m* values and pH dependence to that of PDZ1 in isolation. Under such conditions, it is plausible the intermediate to resemble a structure whereby PDZ1 is folded, whereas PDZ2 is denatured.

**Figure 4:**
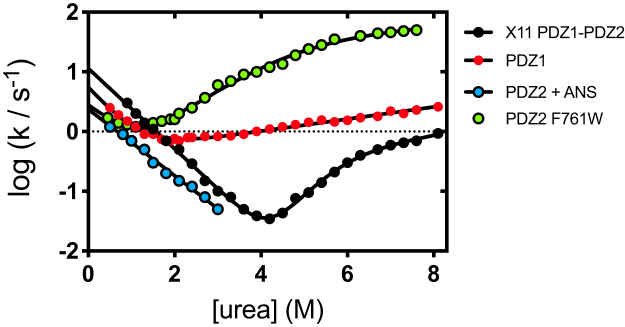
**Folding kinetics of PDZ2 measured by intrinsic and extrinsic fluorescence.** The data are reported in comparison with X11 PDZ1-PDZ2 and PDZ1. The chevron plot obtained for PDZ2 F761W is reported in *green*, whereas the data obtained by extrinsic fluorescence and measured in the presence of ANS on wildtype PDZ2 are reported in *blue*. Of interest, PDZ2 F761W displays an additional roll-over effect indicative of the presence of an additional folding intermediate. Importantly, however, in the case of PDZ2 F761W, analysis with a three-state folding model returns and *m*_D–N_ of 1.2 ± 0.2 kcal mol^−1^ M^−1^, which is consistent with the (un)folding of a single PDZ unit. ANS, 8-anilino-1-naphthalenesulfonic acid.

While the folding kinetics of both X11 PDZ1-PDZ2 and PDZ1 could be readily measured by fluorescence, PDZ2 did not display any appreciable change in signal. To overcome this problem, we resorted to measure its folding both by intrinsic fluorescence, by engineering a fluorescent mutant by mutating position F761 to W, and by extrinsic fluorescence, by performing refolding experiments on wildtype PDZ2 in the presence of 8-anilino-1-naphthalenesulfonic acid (ANS), which selectively binds the native state. The chevron plot of PDZ2-F761W is reported in Figure 4. A comparison of the apparent denaturation midpoint of PDZ2-F761W with the value obtained for wild type PDZ2 by CD equilibrium experiments reveals that insertion of a Trp residue contributes to a destabilization of about 3 kcal mol^−1^, with a midpoint going from 4.1± 0.2 M (wt) to about 1.5 M (PDZ2-F761W). Such destabilization, which may be also observed by comparing the CD-monitored equilibrium denaturation transition (Fig. S3), most likely arises from an increase of the unfolding rate constant. In fact, kinetic experiments conducted on the wildtype protein in ANS returned an essentially identical folding rate constant of 2.6 s^−1^, in the presence of a well characterized refolding arm. This finding indicates that while at this stage, a detailed description of the folding mechanism of PDZ2 cannot be achieved, its folding rate constant in isolation and in the absence of denaturant may be quantified as 2.6 ± 0.3 s^−1^. A typical refolding trace of PDZ2 in the presence of ANS is reported in Fig. S4. Unfortunately, we could not measure unfolding in the presence of ANS as we observed that incubating native PDZ2 with ANS results in a pronounced precipitation of the protein sample. Importantly, this value is lower that both X11 PDZ1-PDZ2 and PDZ1. The implications of these findings are further discussed below.

### A short autoinhibitory C-terminal extension determines the apparent cooperativity of X11 PDZ1-PDZ2

A previous comprehensive characterization of X11 PDZ1-PDZ2 revealed that this protein contains a C-terminal extension that acts as an autoinhibitor segment. In particular, residues Pro 834 to Ile 837, which represent the C-terminal part of the construct, can selectively interact with the binding pocket of PDZ1, thereby blocking its binding capability and modulating its functions (Fig. 1). In an effort to explore the role of the C-terminal extension of X11 PDZ1-PDZ2 in the folding and function of this tandem, we produced a deletion variant of X11 PDZ1-PDZ2, namely X11 PDZ1-PDZ2-ΔC, where we truncated its last nine C-terminal residues.

**Figure 5:**
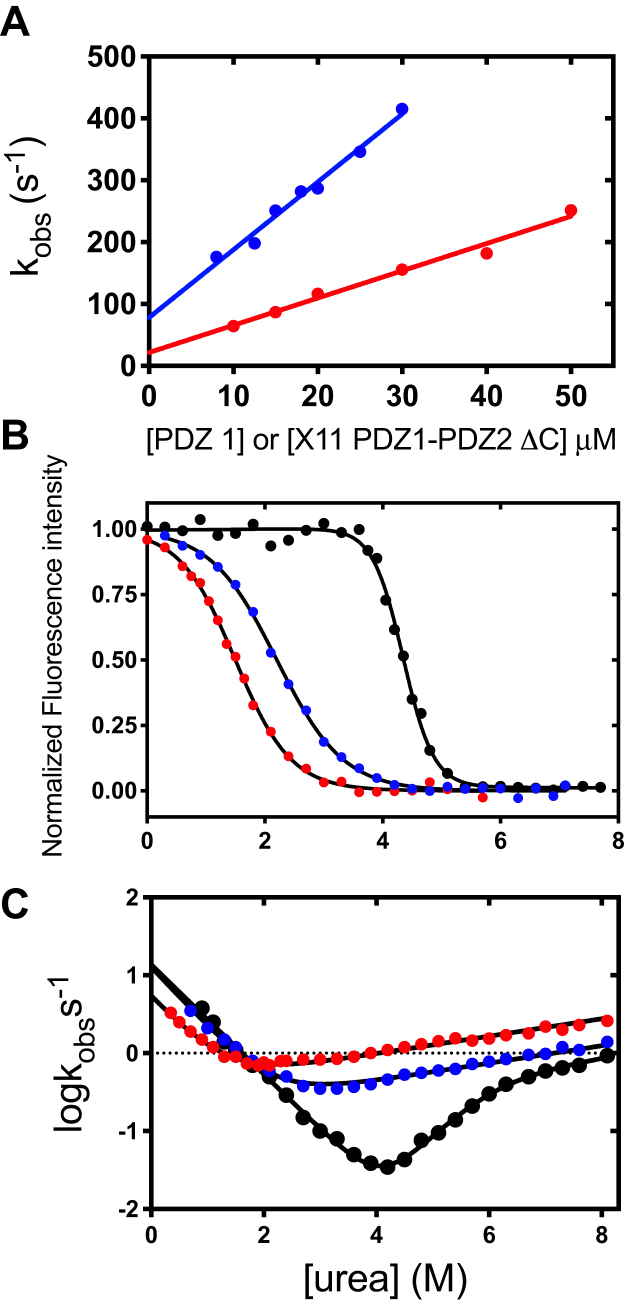
**Folding and binding properties of X11 PDZ1-PDZ2 ΔC.***A*, pseudo-first-order binding plots for PDZ1 (*red*) and X11 PDZ1-PDZ2 ΔC (*blue*). No binding could be observed in the case of X11 PDZ1-PDZ2, further confirming the autoinhibitory role of the C-terminal tail. *B*, fluorescence monitored equilibrium denaturation of X11 PDZ1-PDZ2 ΔC (*blue*) in comparison to X11 PDZ1-PDZ2 (*black*) and PDZ1 (*red*). *Lines* are the best fit to a two-state transition. It is evident that truncation of solely nine amino acids, in the case of X11 PDZ1-PDZ2 ΔC, causes a remarkable decrease in cooperativity that is similar to PDZ1 in isolation*. C*, chevron plot of X11 PDZ1-PDZ2 ΔC (*blue*), in comparison with X11 PDZ1-PDZ2 (*black*) and PDZ1 (*red*). It is evident that deletion of solely nine amino acids decreases the apparent cooperativity with folding kinetics of PDZ1-PDZ2 ΔC closely resembling that observed for PDZ1.

The dansylated AFHQFYI peptide, which also binds selectively to the PDZ1 and mimics the physiological binder preselin-1, was used together with PDZ1, X11 PDZ1-PDZ2, and X11 PDZ1-PDZ2-ΔC in stopped flow binding experiments. In line with the previous suggestions by Zhang *et al.* (21), while we could not observe any binding in the case of X11 PDZ1-PDZ2, both PDZ1 in isolation and X11 PDZ1-PDZ2-ΔC, which both lack the autoinhibitory segment, returned reliable binding time courses. A pseudo first-order plot of the obtained rate constants is reported in Figure 5*A*. Although some changes both in the association and in the dissociation rate constants, which could mirror some shifts in binding mechanism, the apparent *K*_D_ for the two constructs appears very similar being 5.5 ± 2.1 μM for PDZ1 and 7 ± 1 μM for X11 PDZ1-PDZ2-ΔC, further confirming the key role of the C-terminal residues in the autoinhibition of X11 PDZ1-PDZ2.

While the function of the autoinhibitory segment in binding was previously established, it is particularly instructive to investigate its role in stability and folding. The fluorescence-monitored equilibrium transition of X11 PDZ1-PDZ2-ΔC in comparison with X11 PDZ1-PDZ2 and PDZ1 is reported in Figure 5*B*. Interestingly, although the former constructs lack only nine amino acids as compared to the full-length tandem, the observed (un)folding cooperativity is much lower and is very similar to that of PDZ1 in isolation. In fact, the measured *m*_D–N_ value is 1.20 ± 0.04 kcal mol^−1^ M^−1^, as compared to the values of 2.0 ± 0.1 kcal mol^−1^ M^−1^ and 1.30 ± 0.09 kcal mol^−1^ M^−1^ for X11 PDZ1-PDZ2 and PDZ1 respectively. To investigate the role of the C-terminal extension in the folding and stability of PDZ2, we also expressed and characterized this domain in isolation with the truncated last nine C-terminal residues, namely PDZ2-ΔC, and subjected to CD-monitored equilibrium urea induced denaturation. The observed transition is reported in Fig. S2. It is evident that truncation of the last nine residues has only a marginal effect on the folding of the domain, with PDZ2-ΔC returning an apparent *m*_D–N_ value is 1.15 ± 0.07 kcal mol^−1^ M^−1^ and a denaturation midpoint of 3.2 ± 0.1 M, as compared to the respective values of 0.95 ± 0.08 kcal mol^−1^ M^−1^ and 3.9 ± 0.1 M in the case of PDZ2 containing the C-terminal tail.

Additional information on the key role of the autoinhibitory segment in dictating the folding mechanism of X11 PDZ1-PDZ2 comes from the analysis of the folding of X11 PDZ1-PDZ2-ΔC. In fact, the CD-monitored unfolding transition of this construct (Fig. S5) reveals a marked decreased of the observed m value, with an apparent value of 0.63 ± 0.08 kcal mol^−1^ M^−1^. This finding parallels what was previously observed in the case of whirlin, whereby the apparent broad measured transition is the result of the sum of the independent unfolding of the two individual domains. This point is further supported by the chevron plot of X11 PDZ1-PDZ2-ΔC, which is reported in Figure 5*C*. It is evident that, when the C-terminal segment is deleted, folding conforms to the behavior of a single domain with both folding and unfolding rate constants, as well as their respective *m* values, being very similar to PDZ1 in isolation. Importantly, it should be noticed that even if X11 PDZ1-PDZ2-ΔC contains PDZ2, this second domain is optically silent and could not be detected in the time-resolved stopped-flow experiments. On the basis of these observations, it may be concluded that the presence of the C-terminal tail is mandatory to couple the interdependence of the (un)folding of the two contiguous domains, and it is therefore critical in dictating the supradomain cooperativity.

## Discussion

The results described in this work allow analyzing some of the features of cooperative folding in the context of a multidomain system. In fact, the comparison between the folding of X11 PDZ1-PDZ2 to that of its constituent isolated domains highlights a conundrum—while the individual domains are competent for cooperative folding, the underlying mechanism in the multidomain construct significantly differs from expectations and behaves at equilibrium as a single cooperative unit. Notably, a transiently populated folding intermediate may be identified by kinetic analysis of folding, indicating that slide from hierarchical to cooperative folding is subtle and must be closely investigated.

The overall picture arising from the analysis of the different constructs reveal that formation of native structure in PDZ1 is essential in accelerating and stabilizing the folding of PDZ2. On the other hand, the characterization of a truncated variant demonstrates that the presence/absence of the short autoinhibitory C-terminal tail, composed by nine amino acids only, is critical to ensure the cooperative folding of the whole protein. This important observation allows concluding that the long-range interactions between the folded PDZ1 domain and the C-terminal tail exert an essential role both in stabilizing and accelerating the folding of PDZ2, possibly by reducing the conformational space of its denatured state.

One of the most universal features of proteins lies in their folding cooperativity (23). In fact, despite folding is determined by the formation of hundreds of weak noncovalent interactions, protein domains generally reach their native conformation in an all-or-none fashion. In theory, for a given element of substructure to form concurrently with another, two conditions should be satisfied. Firstly, the inherent stability of a given set of interactions must be interdependent on another. Secondly, the rate of formation of individual elements of structure must also be interdependent (1). In such conditions, formation of a folding nucleus (i) stabilizes the remainder of the protein segments, which are therefore more stable in the presence of the nucleus than in its absence and (ii) represents the rate-liming step of folding, whereby emergence of native-like structure in the remainder of the protein is accelerated by the existence of the nucleus. Our results demonstrates that the interdependent folding of the contiguous domains in X11 PDZ1-PDZ2 satisfy these postulates and, therefore, result in a supradomain cooperative transition.

The general manifestation of these phenomena in single domain proteins typically results in the so-called nucleation–condensation mechanism (27, 28, 29). By following this formalism, the formation of a folding nucleus occurs concurrently with a global collapse of the polypeptide chain. The thermodynamic and kinetic independence of the remainder of the protein, which may occur for example by stabilizing individual elements of structure, leads to deviation from this scenario and result in the accumulation of intermediates and hierarchical folding (30, 31). Conversely, we exemplify that, in the case of multidomain systems, a slide from cooperative to hierarchical folding may also occur by abolishing long range interactions, which may abrogate the thermodynamic and kinetic communication between the individual constituent domains. This finding parallels what previously suggested in the case of immunoglobulin domains (1).

The case of X11 PDZ1-PDZ2 represent a rare example whereby a slide from discrete folding of individual domains to cooperative interdependent folding may be captured and successfully explained at a molecular level. Importantly, such transition is mediated by long-range interactions that expand the structural features of the folding nucleus, which, in the folding of the full-length construct, includes the autoinhibitory tail, thereby accelerating the folding of PDZ2. We speculate such long-range effects to be responsible for previous examples of cooperative multidomain folding. Future work on other multidomain domain systems will further test this speculation.

## Experimental procedures

### Proteins mutagenesis, expression, and purification

The coding sequences for X11 PDZ1-PDZ2 (residues 651–837) and X11 PDZ2 (residues 746–837) were obtained from UniProt (code Q02410), synthesized by Eurofins genomics, and cloned in a pET 28b (+) vector. To avoid dimerization in folding and unfolding experiments, cysteines at positions 86, 106, and 133 were all mutated to serine in all constructs. The X11 PDZ1 and X11 PDZ1-PDZ2ΔC constructs were obtained by inserting stop codons in X11 PDZ1-PDZ2 construct in position valine 745 and leucine 828, respectively, or mutagenesis for X11 PDZ2-F761W by using the QuickChange Lightning Site-Directed Mutagenesis kit (Agilent technologies). Proteins were expressed in the *Escherichia Coli* BL-21 (DE3) (BioLabs) strain. Cultures were grown in Luria Bertani medium containing 34 μg/ml kanamycin at 37 °C. After induction with 1 mM IPTG (isopropyl-β-D-thiogalactopyranoside), cells were grown at 22 °C overnight and collected by centrifugation. Pellets were resuspended in 50 mM Hepes pH 7.5, 300 mM NaCl, 10 mM imidazole, and protease inhibitor (Complete EDTA-free, Roche) and sonicated. The supernatant was loaded on a HisTrap FF (GE Healthcare) column equilibrated with the same buffer. Proteins were eluted with an imidazole gradient (10 mM–1M) and collected fractions were buffer exchanged with Hepes pH 7.5, 300 mM NaCl with a HiTrap Desalting column (GE Healthcare). All the constructs in this work contain a N-terminal His tag.

### Equilibrium experiments

CD urea–induced denaturation experiments were performed using a Jasco J710 instrument (Jasco Inc) equipped with a Peltier apparatus for temperature control. Spectra were collected in the far-UV region (200–250 nm) using a quartz cell (1 mm optical path length), and the transition was followed at 222 nm upon urea addition. Protein concentration was 25 μM for all proteins tested. Equilibrium fluorescence measurements of X11 PDZ1-PDZ2, X11 PDZ2, and X11 PDZ1-PDZ2ΔC were performed by following tryptophan intrinsic fluorescence signal at 350 nm (λ_ex_ = 280 nm) as a function of urea concentration, on a FluoroMax- 4 spectrofluorometer (Jobin Yvon) using a 1-cm path length quartz cuvette. The buffer used was for both fluorescence and CD experiments (Hepes 50 mM pH 7.5 at 37 °C). All samples were incuvated for at least 15 min. The urea-induced denaturations were fitted by assuming a two-state mechanism (22).

### Kinetic experiments

Stopped flow experiments were performed on a single-mixing SX-18 instrument (Applied Photophysics) monitoring the change of fluorescence emission. The excitation wavelength used was 280 nm, and the fluorescence emission was recorded by using a cut-off glass filter (320 nm). At least five individual traces were acquired and then averaged for each experiment. All the averages were satisfactorily fitted with a single exponential equation. Experiments were conducted using 3 μM (after mixing) protein sample in 50 mM Hepes pH 7.5 at 37 °C and different concentrations of urea. For pH dependence of X11 PDZ1 and X11 PDZ1-PDZ2, different pH conditions were also tested: Tris HCl pH 8.9, and 8; Hepes pH 7; sodium phosphate pH 6.5; sodium acetate pH 5.5, 5, 4.5 and 3.7. The temperature was 37 °C in all cases.

Extrinsic fluorescence experiment on X11-PDZ2 in the presence of ANS was obtained by mixing denatured X11-PDZ2 in presence of ANS against refolding buffer with ANS (final concentration: [X11-PDZ2] = 3 μM; [ANS] = 300 μM).

X11 PDZ1 and PDZ1-PDZ2ΔC kinetics data were fitted according to a two-state model as follows

(1) $$k_{obs}=k_{f}^{H_{2}O}\;e^{-m_{f}\frac{\left[denaturant\right]}{RT}}+k_{U}^{H_{2}O}\;e^{m_{U}\frac{\left[denaturant\right]}{RT}}$$

where k_f_^H^_2_^O^ and k_u_^H^_2_^O^ are the folding and unfolding rate constants in the absence of denaturant, and *m*_*F*_ and *m*_*U*_ reflect their dependence on denaturant concentration and correlate with the change in accessible surface area between the two ground states and the transition state between them.

In the case of X11 PDZ1-PDZ2 and X11 PDZ2 F761W, a three-state model was used taking into account the presence of an intermediate (25), as follows:

(2) $$k_{obs}=k_{N}^{H_{2}O}\left(-m_{n}\frac{\left[denaturant\right]}{RT}\right)+\frac{\left(〖k_{U2}^{H_{2}O}\;\left(e^{m_{U}2\frac{\left[denaturant\right]}{RT}}\right)\right)}{1+K_{\begin{array}{c} N-I \end{array}}^{H_{2}O}\;\left(e^{m_{KN-I}\frac{\left[denaturant\right]}{RT}}\right)}$$

where K_N–I_ represents the pre-equilibrium constant between the native state at the folding intermediate, and *m*_KN–I_ its associated *m* value.

### Binding experiments

Equilibrium binding experiments between X11 PDZ1, X11 PDZ1-PDZ2, PDZ1-PDZ2ΔC, and Presenilin-1 peptide (AFHQFYI) (GenScript), functionalized with a dansyl group at the N-terminal, were carried out on a FluoroMax- 4 spectrofluorometer (Jobin Yvon) using a 1-cm path length quartz cuvette. Titration experiments were conducted with an excitation wavelength of 280 nm, following intrinsic tryptophan emission at 340 nm. Titrations were performed at constant protein concentration (1.5 μM) and varying peptide concentrations.

Kinetics binding experiments were performed in pseudo-first order condition on a single-mixing SX-18 instrument (Applied Photophysics) by following the FRET signal between Trp residue (donor) in the proteins and dansyl group (acceptor) in the peptide (excitation 280 nm, cut-off filter 475 nm) and at least four independent traces were averaged and satisfactorily fitted to a monoexponential decay. The peptide concentration was maintained fixed at 2 μM (after mixing) while varying the protein concentration. The k_obs_ obtained for binding as a function of protein concentration were fitted with a linear function.

## Data availability

All study data are included in the article.

## Supporting information

This article contains supporting information.

## Conflict of interest

All authors declare no conflict of interest with the contents of this article.
