# Supplementary material for: Understanding the molecular basis of folding cooperativity through a comparative analysis of a multidomain protein and its isolated domains
Source: J Biol Chem. 2023 Feb 3;299(3):102983. doi: 10.1016/j.jbc.2023.102983 (PMC10017356; doi:10.1016/j.jbc.2023.102983)
Supplement: Supplemental Figures S1–S5 [file mmc1.pdf]

SUPPORTING INFORMATION FOR

Understanding the molecular basis of folding cooperativity through a comparative analysis of a multidomain protein and its isolated domains

Daniele Santorelli[1], Lucia Marcocci[1], Valeria Pennacchietti[1], Caterina Nardella[1], Awa Diop[1], Paola Pietrangeli[1], Livia Pagano[1], Angelo Toto[1], Francesca Malagrinò[2,]* and Stefano Gianni[1,*]

[1]Istituto Pasteur-Fondazione Cenci Bolognetti and Istituto di Biologia e Patologia Molecolari del CNR, Dipartimento di Scienze Biochimiche "A. Rossi Fanelli," Sapienza Università di Roma, 00185 Rome, Italy.

[2]Dipartimento di Farmacia, Università degli Studi di Napoli Federico II, Naples, Italy

* = Corresponding Authors: Francesca Malagrinò (francesca.malagrino@unina.it) Stefano Gianni (stefano.gianni@uniroma1.it)

Running title : The folding of a PDZ tandem repeat

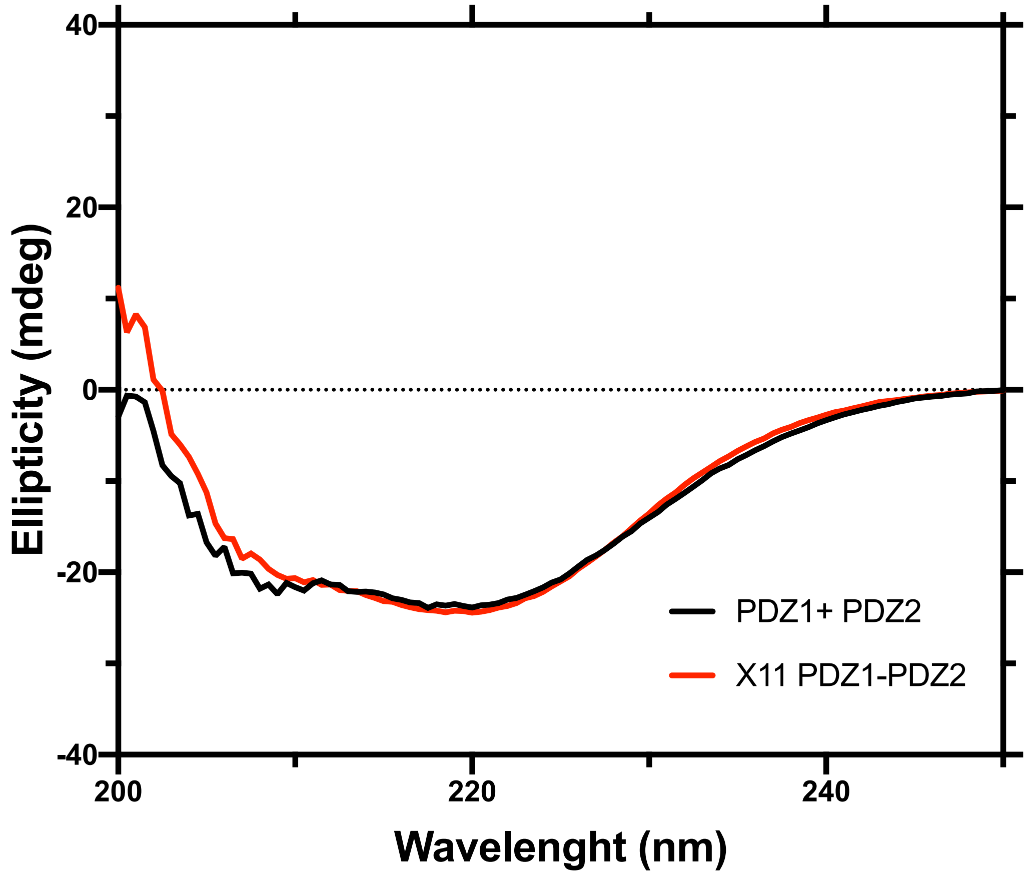

Figure S1. Comparison of the CD spectra of X11 PDZ1-PDZ2 (red) and its isolated constituent domains (black). To verify that the overall structure of PDZ1 and PDZ2 was not substantially perturbed by expressing them in isolation, the CD spectrum of X11 PDZ1-PDZ2 was compared with a solution containing equimolar concentration of both domains. Data were recorded at protein concentration of 25 μM in Hepes 50 mM pH 7.5 at 37°C.

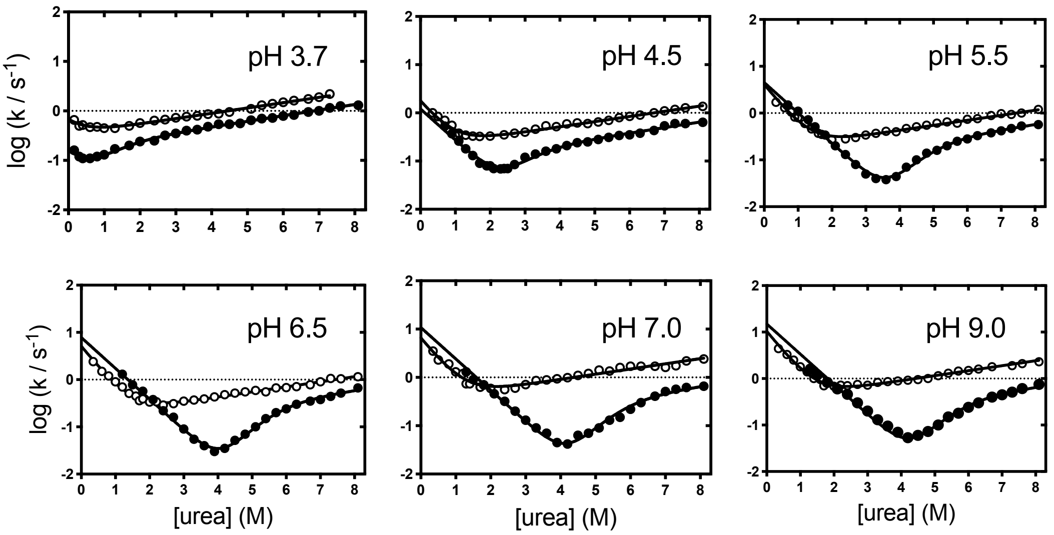

Figure S2. Chevron plot for X11 PDZ1-PDZ2 and PDZ1 recorded at different pH conditions. The data referring to X11 PDZ1-PDZ2 and PDZ1 are reported in filled and open circles respectively. In the case of X11 PDZ1-PDZ2 data were fitted to a three state model as described in the experimental procedure section. Whereas PDZ1 could be well fitted to a two state model. The buffer used are described in the experimental procedure section.

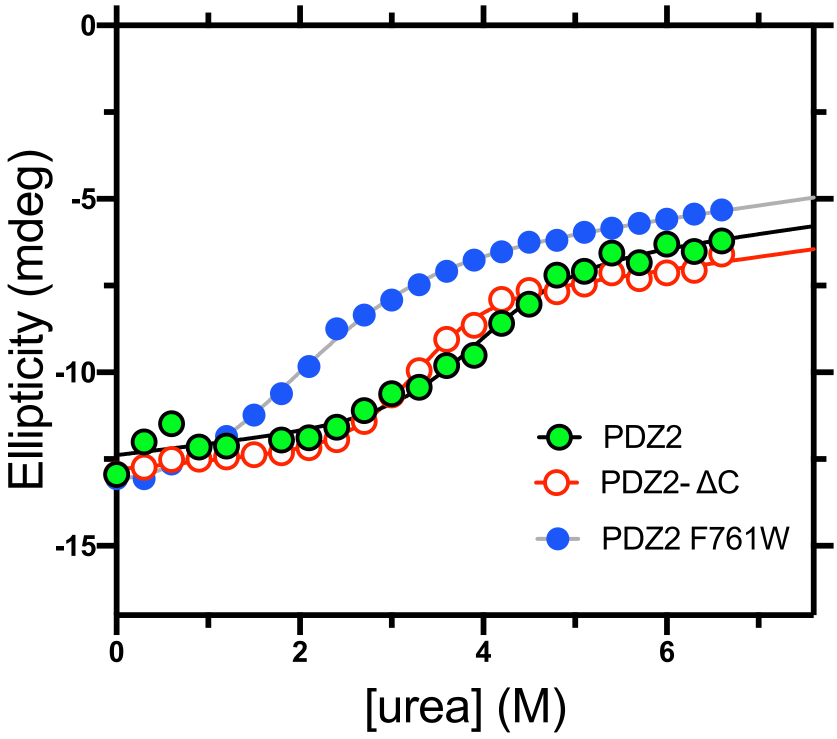

Figure S3. CD monitored equilibrium denaturation of PDZ2 (green), PDZ2-ΔC (red) and PDZ2 F761W (blue). Lines are the best fit to a two-state equilibrium unfolding transition. Data were recorded at protein concentration of 25 μM in Hepes 50 mM pH 7.5 at 37°C.

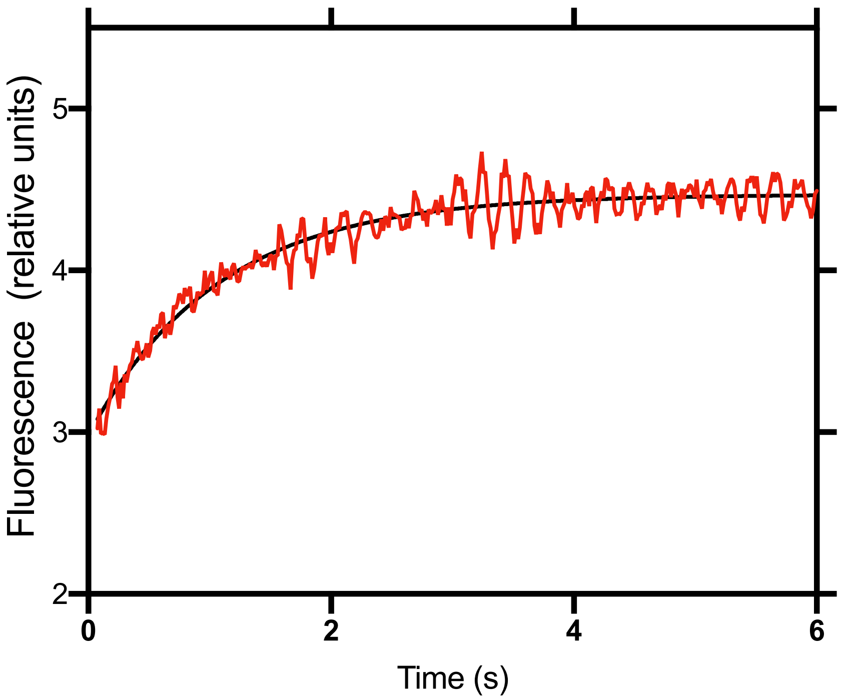

Figure S4. Typical folding time course of PDZ2 measured by extrinsic fluorescence in the presence of ANS. The reported folding time course, which was obtained by averaging 6 independent traces, were recorded in the presence of a final urea concentration of 0.6 M. The solid black line is the best fit to a single exponential decay.

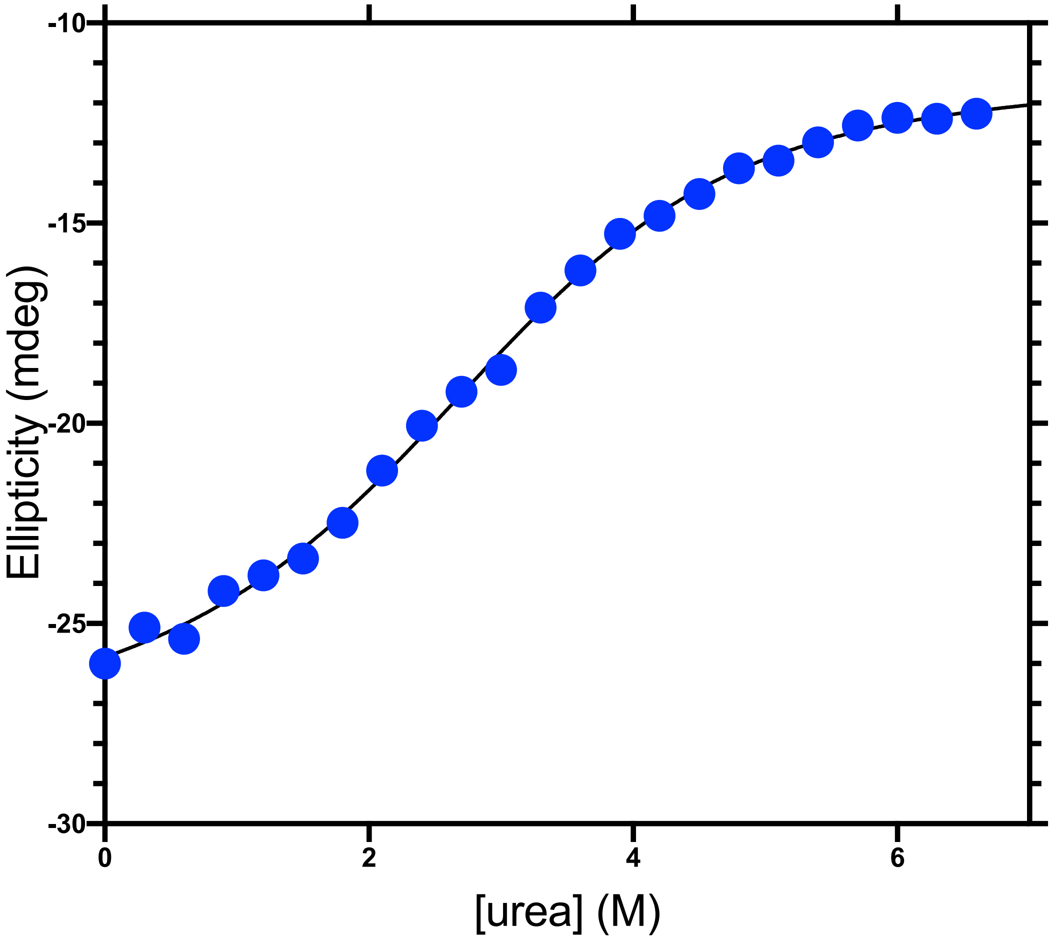

Figure S5. CD monitored equilibrium denaturation of PDZ1-PDZ2-ΔC. The line is the best fit to a two-sate equilibrium transition. In analogy with what previously observed in the case of the PDZ tandem of whirlin, as expected if when the two contiguous domains unfold independently on each other, we observed a broader transition with an apparent lower *m*-value ($m_{D\text{-}N}$ = 0.63 ± 0.06 kcal mol^{-1} M^{-1}) that simply represents the sum of the two individual denaturation curves.
